# Impact of deep learning denoising on kinetic modelling for low-dose dynamic PET: application to single- and dual-tracer imaging protocols

**DOI:** 10.1007/s00259-025-07182-6

**Published:** 2025-03-12

**Authors:** Florence M. Muller, Elizabeth J. Li, Margaret E. Daube-Witherspoon, Austin R. Pantel, Corinde E. Wiers, Jacob G. Dubroff, Christian Vanhove, Stefaan Vandenberghe, Joel S. Karp

**Affiliations:** 1 Medical Image and Signal Processing, Faculty of Engineering and Architecture, Ghent University, Ghent, Belgium; 2 Physics and Instrumentation, Department of Radiology, University of Pennsylvania, Philadelphia, PA US; 3 Division of Nuclear Medicine Imaging and Therapy, Department of Radiology, University of Pennsylvania, Philadelphia, PA US; 4 Center for Studies of Addiction, Department of Psychiatry, University of Pennsylvania, Philadelphia, PA US

**Keywords:** Deep learning denoising, Dynamic LAFOV PET, Low-dose imaging, Dual-tracer protocols

## Abstract

**Purpose:**

Long-axial field-of-view PET scanners capture multi-organ tracer distribution with high sensitivity, enabling lower dose dynamic protocols and dual-tracer imaging for comprehensive disease characterization. However, reducing dose may compromise data quality and time-activity curve (TAC) fitting, leading to higher bias in kinetic parameters. Parametric imaging poses further challenges due to noise amplification in voxel-based modelling. We explore the potential of deep learning denoising (DL-DN) to improve quantification for low-dose dynamic PET.

**Methods:**

Using 16 [^18^F]FDG PET studies from the PennPET Explorer, we trained a DL framework on 10-min images from late-phase uptake (static data) that were sub-sampled from 1/2 to 1/300 of the counts. This model was used to denoise early-to-late dynamic frame images. Its impact on quantification was evaluated using compartmental modelling and voxel-based graphical analysis for parametric imaging for single- and dual-tracer dynamic studies with [^18^F]FDG and [^18^F]FGln at original (injected) and reduced (sub-sampled) doses. Quantification differences were evaluated for the area under the curve of TACs, K_i_ for [^18^F]FDG and V_T_ for [^18^F]FGln, and parametric images.

**Results:**

DL-DN consistently improved image quality across all dynamic frames, systematically enhancing TAC consistency and reducing tissue-dependent bias and variability in K_i_ and V_T_ down to 40 MBq doses. DL-DN preserved tumor heterogeneity in Logan V_T_ images and delineation of high-flux regions in Patlak K_i_ maps. In a /[^18^F]FDG dual-tracer study, bias trends aligned with single-tracer results but showed reduced accuracy for [¹⁸F]FGln in breast lesions at very low doses (4 MBq).

**Conclusion:**

This study demonstrates that applying DL-DN trained on static [^18^F]FDG PET images to dynamic [^18^F]FDG and [^18^F]FGln PET can permit significantly reduced doses, preserving accurate FDG K_i_ and FGln V_T_ measurements, and enhancing parametric image quality. DL-DN shows promise for improving dynamic PET quantification at reduced doses, including novel dual-tracer studies.

**Supplementary Information:**

The online version contains supplementary material available at 10.1007/s00259-025-07182-6.

## Introduction

In clinical practice, most positron emission tomography (PET) scans are acquired at a single time-point, about 60 min post-injection (p.i.) for [^18^F]FDG, using standardized uptake values (SUV) to reflect the total tissue activity at that particular time-point. However, tracer distribution is a dynamic and tracer-specific process that varies across organs and patients [1]. With dynamic PET, temporal changes in tracer uptake can be assessed by measuring time-activity curves (TACs), allowing kinetic analysis to differentiate between specific (pathological) and non-specific (physiological) signals [1–3]. Parameters quantifying tracer delivery measured by the blood-to-tissue transport constant (K_1_) and flux (K_i_) can provide insights into drug delivery and substrate uptake processes, outperforming static SUV measures [4–6]. Kinetic analysis of dynamic PET usually involves the reconstruction of multi-frame images, starting with short (typically 5–15 s) time frames after injection to capture the rapid uptake and the blood input function (IF), followed by longer (typically 1–5 min) time frames to measure tracer wash-out from the tissue. However, short frames suffer from increased noise due to low-count statistics, impacting TAC fitting and IF accuracy and contributing to higher biases and lower precision in kinetic parameters [2, 7, 8]. Reduced statistical quality becomes even more pronounced when aiming for low dose dynamic PET. Increased noise in the data exacerbates the reliability of kinetic parameter quantification at both the organ and voxel level. There is a push for lower dose protocols to reduce radiation exposure or allow the administration of different radiotracers for further disease characterization. To enable multiple PET scans, in dual- or multi-tracer studies, it is essential to reduce the injected doses while optimizing delivery protocols and minimizing cross-talk [8–12].

The development of long axial field-of-view (LAFOV) PET systems has resulted in an increased system sensitivity and allowed for dynamic imaging of the complete time-course of a tracer across a large axial FOV [13, 14]. With these sensitivity advantages, LAFOV PET can accurately quantify tracer kinetics in multiple tissues and offer new possibilities to make dynamic PET more robust and feasible for clinical use [15–17]. There is, though, potential to further enhance dynamic protocols and facilitate the translation to standard AFOV PET systems. Several reconstruction and image processing techniques have been investigated to improve 4D PET imaging [10, 11], including dynamic image reconstruction algorithms [18–21] and post-reconstruction denoising approaches [22, 23]. Parametric reconstruction algorithms to directly estimate kinetic parameters have also been developed for both linear and non-linear kinetic models [10, 11], and recently deep learning (DL) approaches for direct and fast parametric reconstruction have gained significant attention [24–26]. DL-based methods incorporating the Deep Image Prior (DIP) have been applied post-reconstruction to denoise dynamic PET images [27–29]. For example, Hashimoto et al. have proposed 3D [27] and 4D [28] DIP models. Cheng et al. [29] proposed to use two 2D CNN-based DIP architectures to simultaneously denoise all dynamic images.

This study explores image denoising for dynamic PET by utilizing a DL framework trained on [^18^F]FDG PET images acquired at a late time-point for a duration that aligns with clinical scan protocols (i.e., a 10-min scan at 50 min p.i.). These static images, sub-sampled to several count reduction factors, are used for training separate denoising neural networks. This work assesses the impact of DL-denoising (DL-DN) on kinetic parameter quantifications derived from compartmental modelling and parametric imaging using voxel-based graphical analysis of low-dose dynamic [^18^F]FDG and [^18^F]fluoroglutamine ([^18^F]FGln) PET. We present kinetic results from dynamic single-tracer scans, assessing the net influx rate of tissues for [^18^F]FDG and the volume of distribution of breast cancer lesions for [^18^F]FGln. We also investigate the potential of DL-DN for low-dose dual-tracer imaging in a single-imaging session with [^18^F]FGln and [^18^F]FDG in breast cancer patient to provide complementary measures of glutaminolysis and glycolysis, respectively [30, 31].

## Materials and methods

### Datasets

Eighteen [^18^F]FDG research subjects from two protocols (Table 1) were dynamically scanned on the PennPET Explorer (142 cm AFOV [32]). These protocols provide varied FDG distributions over a range of patient BMI for both training and testing our DL-DN model. 5 datasets were collected from subjects with epilepsy (all used for training). 13 datasets (11 for training and 2 for testing) were obtained from a pilot study [33] investigating the pharmacokinetic effects of nutritional ketone ester (KE) on brain ketone and glucose metabolism in alcohol use disorder (AUD). Among these, 5 participants (3 healthy controls (HC), 2 with AUD) underwent two [^18^F]FDG PET scans; one following KE intervention (where the KE reduces brain glucose metabolism by ∼20% and suppresses cardiac uptake) and one at baseline (without KE), resulting in 10 datasets. Additionally, 3 participants only completed one [^18^F]FDG PET scan: 1 HC (baseline scan) and 2 subjects with AUD (with KE).

**Table 1:** Overview of the datasets and scan protocol characteristics used for training and testing

	Datasets from the pilot study on alcohol use disorder [33]	Datasets from subjects with epilepsy
**Total number of studies**	13	5
• Training	11	5
• Testing	2	0
**10-min data (training)**	50–60 min p.i.	90–100 min p.i.
**Dynamic data (testing)**	0–60 min p.i.	-
**BMI (kg/m** ^**2**^ **)**	26.1 ± 5.5	32.4 ± 10.7
**[** ^**18**^ **F]FDG dose (MBq)**	378 ± 26	380 ± 27

#### Training

From the 16 training studies, we extracted the final 10-min to reflect delayed static images. For the subjects enrolled in the pilot study on AUD, data were extracted from 50 to 60 min p.i., and for epilepsy subjects, from 90 to 100 min p.i. These 10-min datasets were used as the target training image. The 10-min list-mode data were sub-sampled with event shuffling and splitting to generate list-files with 1/2, 1/5, 1/10, 1/20, 1/60, 1/120 and 1/300 of the original counts.

#### Testing

##### Single-tracer [^18^F]FDG

Performance of the DL-DN model was evaluated using the 60-min dynamic [^18^F]FDG data from ten subjects in the pilot study on AUD. Note that the same cohort of subjects was used for both training and testing; since only their late time-point data (50–60 min p.i.) were used for training, the dynamic data remained appropriate for testing. The dynamic data were parsed into 39 frames: 12 × 5s, 6 × 10s, 3 × 20s, 2 × 30s, 6 × 1 min, 10 × 5 min. To emulate low-dose dynamic [^18^F]FDG studies, each frame was sub-sampled to 1/2, 1/5, 1/10 and 1/20 of the counts with event shuffling and splitting.

##### Single-tracer [^18^F]FGln

A subject with estrogen-receptor positive (ER+) breast cancer (body mass index (BMI) 28.5 kg/m^2^) was injected with 260 MBq of [^18^F]FGln and dynamically imaged for 70-mins on the 112-cm PennPET Explorer (5-ring system). To replicate the [^18^F]FGln set-up from the dual-tracer study described below in **(c)**, only the first 30-mins of data were considered for subsequent analyses. The dynamic data were divided into 44 frames: 24 × 5s, 6 × 10s, 3 × 20s, 2 × 30s, 5 × 1 min, 4 × 5 min. Previous work [30, 31] demonstrated that the kinetic parameters of interest can be reliably quantified for scan durations as short as 30 min. In this work, the 30-min dynamic data were further sub-sampled with event shuffling and splitting to equivalent doses of 40, 20, 8 and 4 MBq [^18^F]FGln.

##### Dual-tracer [^18^F]FGln/[^18^F]FDG

A subject with ER + breast cancer (BMI: 39.3 kg/m^2^) was first injected with 40 MBq [^18^F]FGln and scanned for 29 min on the PennPET Explorer. At 30 min p.i. of [^18^F]FGln, the subject was injected with 400 MBq [^18^F]FDG and scanned for an additional 65 min. This dual-tracer protocol leveraged the high sensitivity of the PennPET Explorer to use a lower injected dose of [^18^F]FGln, with no loss of accuracy [30, 31], followed by a higher dose of [^18^F]FDG to eclipse the residual [^18^F]FGln signal when modelling the [^18^F]FDG. [^18^F]FGln data were divided into 33 frames: 12 × 5s, 6 × 10s, 3 × 20s, 2 × 30s, 6 × 1 min, 3 × 5 min, 1 × 4 min. [^18^F]FDG data were split into 40 frames: 12 × 5s, 6 × 10s, 3 × 20s, 2 × 30s, 6 × 1 min, 11 × 5 min. The dual-tracer data were sub-sampled with event shuffling and splitting to emulate dose levels of 20, 8 and 4 MBq for [^18^F]FGln as well as 80 and 40 MBq for [^18^F]FDG.

#### Image reconstruction

Images were reconstructed into 4 × 4 × 4 mm^3^ voxels using a list-mode time-of-flight ordered-subsets expectation-maximization algorithm (TOF-OSEM, 5 iterations and 25 subsets) that incorporates spherical image basis functions instead of cubic voxels [34, 35]. The following data corrections were applied: CT-based attenuation correction, normalization correction [14], delays-based randoms correction, and TOF-enhanced single scatter simulation for scatter estimation [36]. For the dynamic data, each frame was reconstructed using the same list-mode TOF-OSEM algorithm, but, for the reconstructions of sub-sampled (lower count) images, the scatter and randoms corrections were not regenerated: the correction files from the original full-dose frames were scaled to the sub-sampled data to ensure quantitative consistency. This scaling approach is appropriate for both early and late phases since list-mode shuffling was used prior to sub-sampling each frame. By randomly sampling events across frames to emulate low-dose dynamic scans, we avoided dependence on specific tracer kinetics within a frame, where temporal changes might occur.

### Deep learning denoising model

#### Framework

**Fig. 1:**
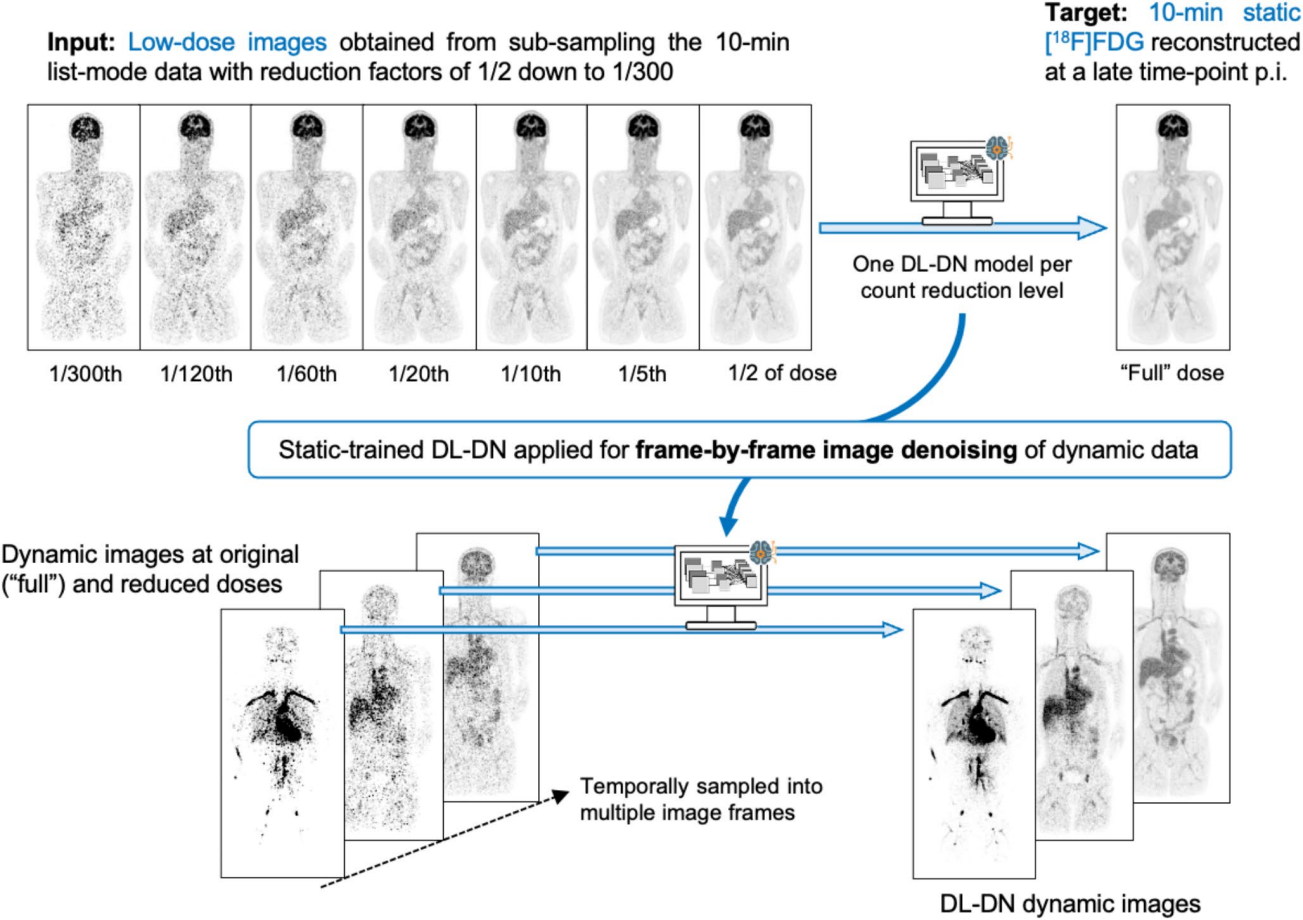
Schematic of the DL-based framework trained on static, single-frame [^18^F]FDG PET images from late phase of uptake for denoising dynamic (= multi-frame) PET images. Static 10-min data were sub-sampled from 1/2 to 1/300 of the counts to train seven denoising networks for each count level studied. The trained DL-DN model was used for frame-by-frame image denoising in dynamic PET at original and reduced doses

We present a method for denoising dynamic PET images using a DL model trained on static [18F]FDG PET images reconstructed at a late time-point p.i. These single-frame images were sub-sampled with seven reduction factors (1/2, 1/5, 1/10, 1/20, 1/60, 1/120, and 1/300 of the original counts) to train seven supervised denoising neural networks for each count level studied. Our adopted training strategy (Fig. 1) leverages this dataset of static images at multiple count reduction factors to develop a model capable of effectively handling the variability in count statistics across dynamic time frames.

The resultant “static-trained” DL-DN model was used for frame-by-frame denoising of dynamic PET data, which were reconstructedas multiple frames at different time-points and of varying durations. Each frame was denoised by the network trained on the static condition that most closely matched its count statistics (see Suppl. Material (1) for additional details). The sub-sampled dose levels used for network training adequately encompassed the range of count statistics observed in the dynamic studies. It is important to note that this framework focused on the total counts in the global image, without accounting for temporal variations in uptake profiles nor adjusting for count density differences. For example, early frames have reduced counts owing to short duration with the activity concentrated in blood-containing structures (e.g., heart, blood vessels), while for later time frames the total activity is more diffusely spread throughout the body; these differences were ignored in the current DL-DN model.

#### Network structure

A 2D U-Net based convolutional neural network (CNN) [37] with attention gates was implemented, featuring 18 2D convolutional layers, three max pooling layers, and three bilinear up-sampling operations (see Suppl. Figure 2). The network processed three consecutive image slices as input channels to generate predictions for the middle slice, capturing spatial context and accounting for relationships between adjacent slices. Batch normalization and parametric ReLU activation function [38] were used. Skip connections [39, 40] and self-attention gating modules (described by Oktay et al. [41]) were integrated into the U-Net architecture. Because attention gates work by assigning weights to different spatial locations in input, a larger receptive field was enabled for the network to selectively prioritize regions that are critical for preserving relevant features while filtering out noise. A residual connection between the input and output was added to enforce for the network to learn the residual function between the noisy low-count input image and the higher-count target image. The number of trainable parameters was approximately 11.2 million.

#### Training and optimization

Network training used 2D transverse, coronal, and sagittal slices normalized by their respective means of each individual input slice with image values expressed in SUV (normalized to body weight). To incorporate volumetric information, the 3D image data were resorted into these three views, transforming it into three sequences of 2D images, as also described in [42]. This approach allowed the network to process the entire 3D dataset efficiently by handling full slices end-to-end, without the need for a sliding-window (patching) method, which would otherwise be necessary due to the computational demands of 3D network inputs. Final predictions were obtained by applying the model separately along all the three views and then averaging the resulting image volumes. A pixel-wise loss function using the mean squared error and the Adam optimizer [43] were implemented with a learning rate of 1E-5 and a batch size of 32.

### Performance evaluations

Volumes of interest (VOIs) were drawn on the original (“full”) dose non-denoised data using the VOI definition tools in PMOD V4.4. (PMOD Technologies LLC, Zurich, Switzerland), and applied to the sub-sampled data.

#### [18F]FDG kinetic modelling

From the 10 dynamic [^18^F]FDG test datasets, tissue TACs were obtained for liver, lung, psoas muscle, and thalamus. An image-derived IF (IDIF) was measured from the descending aorta (DA) for the liver, muscle, and thalamus, and from the right ventricle (RV) for the lung. To assess differences in TACs between non-denoised and DL-DN at various doses, the percent bias in the area under the curve (AUC) of all IDIFs and tissue TACs was calculated relative to the full-dose non-denoised reference.

The [^18^F]FDG TACs were fit using a 2-tissue-compartment model (2-TCM) with irreversible tracer uptake (k_4_ = 0) and with considerations of blood delay and blood volume. The non-hepatic regions were fit using the PKIN tool in PMOD V4.4. For liver kinetic modelling, an optimization-derived dual-blood IF model implemented in MATLAB [44] was used to account for the blood supply from both the hepatic artery and portal vein. The net influx rate of [^18^F]FDG, K_i_ (ml⋅min^− 1^⋅cm^− 3^), was estimated as [45]: $\:{\text{K}}_{\text{i}}=\frac{{\text{K}}_{1}\:\bullet\:{\text{k}}_{3}}{\left({\text{k}}_{2}+{\text{k}}_{3}\right)}$ where K_1_ (ml⋅min^−1^⋅cm^−3^) represents the uptake rate from the blood into the cell, k_3_ (min^−1^) the rate of phosphorylation, and k_2_ (min^−1^) the clearance rate of unphosphorylated [^18^F]FDG into the blood pool. The percent bias in K_i_ relative to the full-dose non-denoised reference was calculated for both non-denoised and DL-DN data at various dose levels.

The bias % in AUC of TACs and in K_i_ was reported as mean and standard deviation (SD) over the 10 [^18^F]FDG test subjects. Pairwise comparisons between non-denoised and DL-DN at all dose levels were conducted using two-tailed paired sample t-tests, with Bonferroni correction and applying a significance level of 0.01.

Patlak graphical analysis [46] was also performed to estimate the influx rate K_i_ of [^18^F]FDG (K_i_ = Patlak slope) where the tracer is assumed to be irreversibly trapped. K_i_ images were created with an IDIF from the DA and an equilibrium time t* of 25 min. The Patlak-based K_i_ images generated for the 10 [^18^F]FDG test subjects were used to report a mean and SD in root mean square error (RMSE) and structural similarity index metric (SSIM) computed for the 3D image with respect to the full-dose non-denoised image.

#### [18F]FGln kinetic modelling

The IDIF was measured from the DA, and we defined the lesion as 60% of the maximum voxel value in a larger VOI covering the primary tumor volume. This threshold was chosen because it generated more consistent TAC profiles compared to the standard definition (of 1cm^3^ SUV_peak_ VOI), particularly for this lesion, which had a necrotic center and tumor heterogeneity. The lesion TAC was fit using a 1-tissue-compartment model (1-TCM) with a DA-derived IDIF and considerations of blood delay and blood volume. Glutamine pool size, measured by the volume of distribution (V_T_) [ml⋅cm^− 3^] of [^18^F]FGln, was estimated as: $\:{\text{V}}_{\text{T}}=\frac{{\text{K}}_{1}}{{\text{k}}_{2}}$. To evaluate differences between non-denoised and DL-DN across dose levels, the bias % in AUC of TACs and in V_T_ was quantified relative to the full-dose non-denoised reference. Logan graphical analysis [47] was also performed to generate a parametric V_T_ image with an equilibrium time t* of 2 min. Images were assessed using RMSE and SSIM with respect to the full-dose non-denoised image.

#### Dual-tracer [18F]FGln/[18F]FDG kinetic modelling

The lesion VOI was defined using the previously described 60% threshold within a larger VOI covering the primary tumor to account for heterogeneity. A DA-IDIF was used to estimate the V_T_ of [^18^F]FGln with a 1-TCM, while K_i_ of [^18^F]FDG was derived from a 2-TCM (k_4_ = 0). For variability estimations, list-mode bootstrapping [48, 49] of the dual-tracer data generated 10 statistical replicates per frame for all dose datasets of both tracers. The bias % in the TAC AUC and kinetic parameters (V_T_, K_i_) was reported as mean and SD across bootstrapped datasets. Two-tailed paired sample t-tests, with Bonferroni correction and significance level of 0.01, compared non-denoised and DL-DN at all doses. Parametric images were generated for both tracers; V_T_ images were obtained by Logan analysis of [^18^F]FGln (t* = 2 min), and K_i_ images by Patlak analysis of [^18^F]FDG (t* = 25 min). The image quality of the parametric images was assessed using RMSE and SSIM with respect to the full-dose non-denoised images.

## Results

### [18F]FDG kinetic modelling

**Fig. 2:**
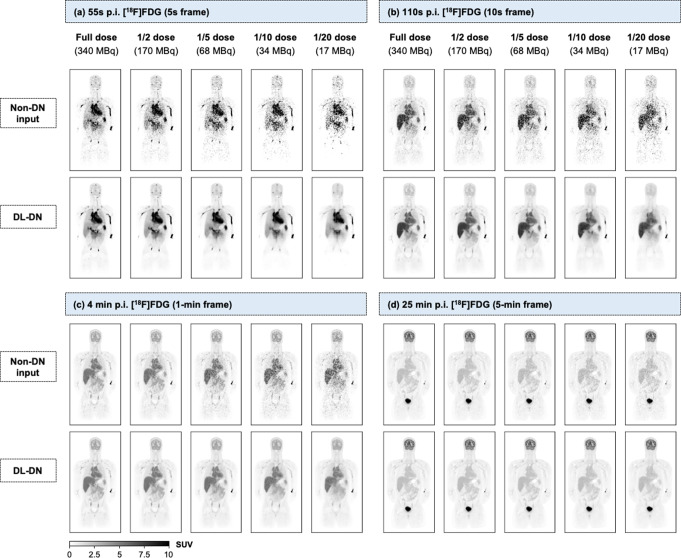
Comparison of a representative 4-mm coronal slice (BMI: 20.9 kg/m^2^) at five doses (columns) between non-denoised (non-DN) and DL-DN images (rows) for four time-points p.i. of a 60-min [^18^F]FDG dynamic scan: (**a**) 55–60 s, (**b**) 110–120 s, (**c**) 4–5 min, and (**d**) 25–30 min

Figure 2 shows a coronal [^18^F]FDG slice reconstructed at 55–60 s, 110–120 s, 4–5 min and 25–30 min p.i. for a subject with AUD following KE administration. These four time-points represent varied activity distributions of different frame durations and count statistics. The dynamic images are compared at different dose levels, each processed post-reconstruction with DL-DN, which effectively reduces noise in the [^18^F]FDG image frames. Without denoising, image quality deteriorates significantly with lower doses. Figure 2 suggests that DL-DN maintains visual quality across dose levels, while noting a slight loss of contrast and blurrier tissue boundaries at 1/20 dose. Lower-dose images for shorter frames (55–60 s, 110–120 s p.i.) exhibit increased graininess in noise texture, leading to a progressive loss of structural integrity. For these early time points, DL-DN shows notable improvements at 1/2 and 1/5 dose, while for longer frames (4–5 min, 25–30 min), its benefits are more pronounced at 1/10 and 1/20 dose.

**Fig. 3:**
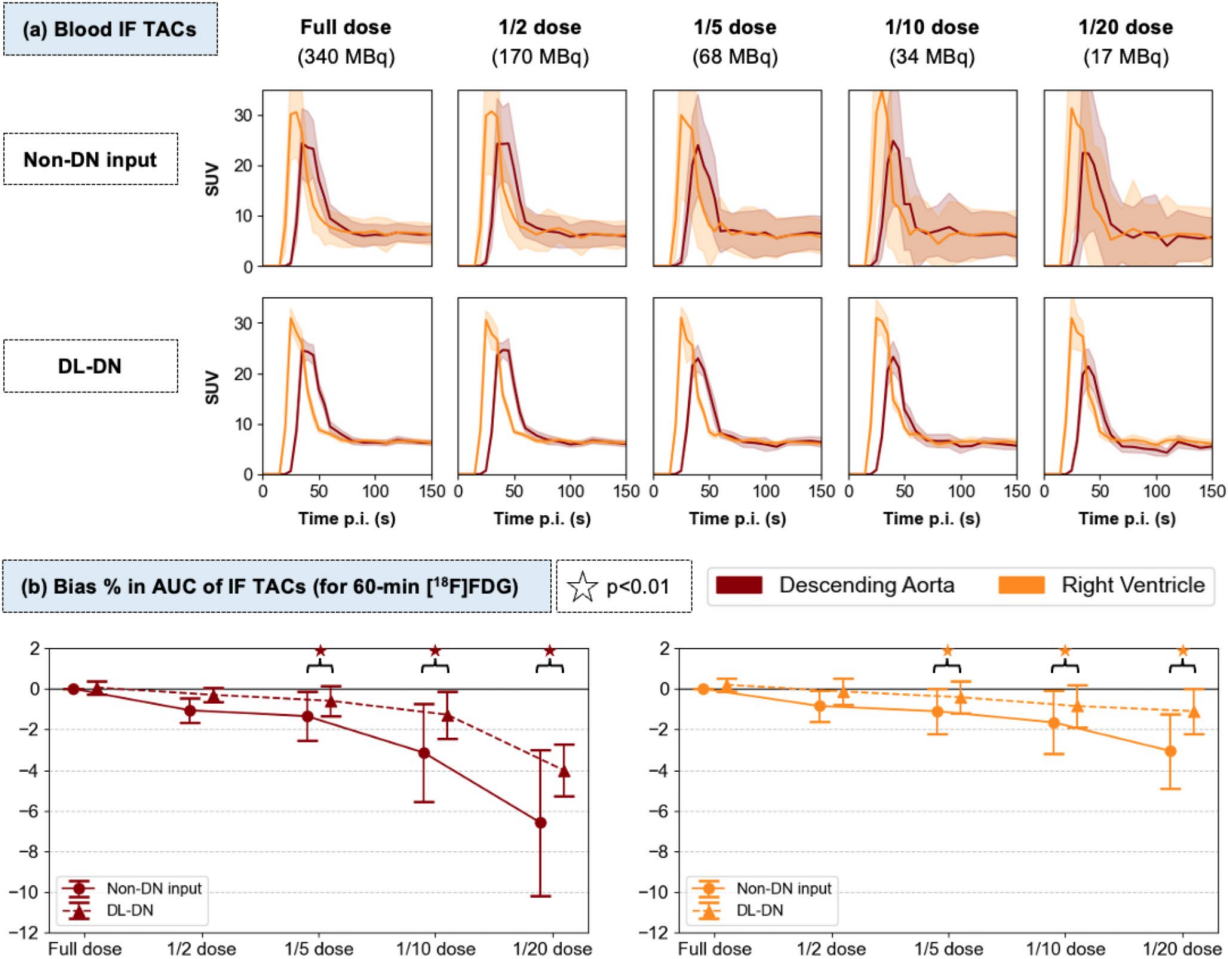
**(a)** Comparison of TACs for image-derived blood IFs measured in the descending aorta (red) and right ventricle (orange) from a single [^18^F]FDG subject. TACs are shown for five doses, comparing non-DN and DL-DN (with slight off-set for visual clarity). Solid lines represent SUV_mean_ in the VOI and shaded areas indicate the SD in SUV. **(b)** Percent bias in AUC for the 60-min IF TACs relative to the full-dose non-DN data, reported as mean and SD over 10 [^18^F]FDG test subjects. Statistical significance (*p* < 0.01) is shown for pairwise comparisons of non-DN and DL-DN results

**Fig. 4:**
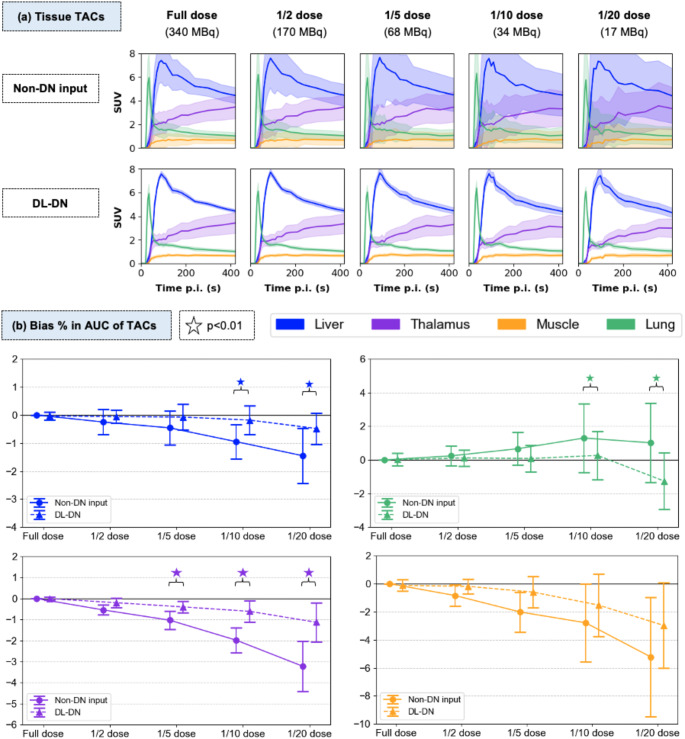
**(a)** Comparison of TACs for four tissues (liver in blue, lung in green, thalamus in purple, muscle in orange) from a single [^18^F]FDG subject. TACs are shown for five doses, comparing non-DN and DL-DN (with slight off-set for visual clarity). Solid lines represent SUV_mean_ in the VOI and shaded areas indicate the SD in SUV. **(b)** Percent bias in AUC for the 60-min tissue TACs relative to the full-dose non-DN data, reported as mean and SD over 10 [^18^F]FDG test subjects. Statistical significance (*p* < 0.01) is shown for pairwise comparisons of non-DN and DL-DN

Figures 3a and 4a present TACs derived from [^18^F]FDG kinetics for blood IFs (DA, RV) and various tissue regions, including liver, thalamus, muscle, and lung. Lower doses result in increased variability, particularly in the non-denoised input data, as reflected by the shaded areas seen on the TACs that represent the SD of SUV measure in the VOI. DL-DN markedly reduces the SD seen in the TACs. Qualitatively, the overall morphology of the TACs is preserved across all dose levels and after DL-DN (e.g., note the RV peak before the DA peak). Compared to the non-denoised data, the DL-DN TACs more closely align with the full-dose non-denoised reference across all sub-sampled doses, as quantified by Figs. 3b and 4b, which show the bias in AUC for the IF and tissue TACs, respectively. For the bias in the AUC of the IF TACs (Fig. 3b), significant differences between non-denoised and DL-DN were observed for doses below 1/5 of full-dose (i.e., < 70 MBq), with considerably lower variability. Notably, the DA shows a steeper downward trend at 1/10 and 1/20 doses compared to the RV, likely due to a reduced peak amplitude of the DA, as exemplified for one test subject in Fig. 3a.

Figure 4b quantifies the bias % in AUC for the four tissue TACs. The liver, thalamus and muscle exhibit similar trends, of increasing negative biases and greater SD as dose levels decrease. DL-DN effectively mitigates both bias and variability. Significant differences were observed between non-denoised and DL-DN starting at 1/10 dose for the liver and 1/5 dose for the thalamus. In contrast, the lung shows a small (< 2%) positive bias across all dose levels, except for the DL-DN at 1/20 dose. This difference in trend may be attributed to the sharp peak in lung TACs within the first minute, reflecting rapid uptake and clearance, whereas other regions exhibit a more gradual uptake or clearance pattern. Notably, variability (SD in % bias) is greater in low-uptake regions, such as the lung and muscle. While no significant differences between non-denoised and DL-DN TACs are observed for the muscle, the lung shows significant differences below the 1/10 dose level.

**Fig. 5:**
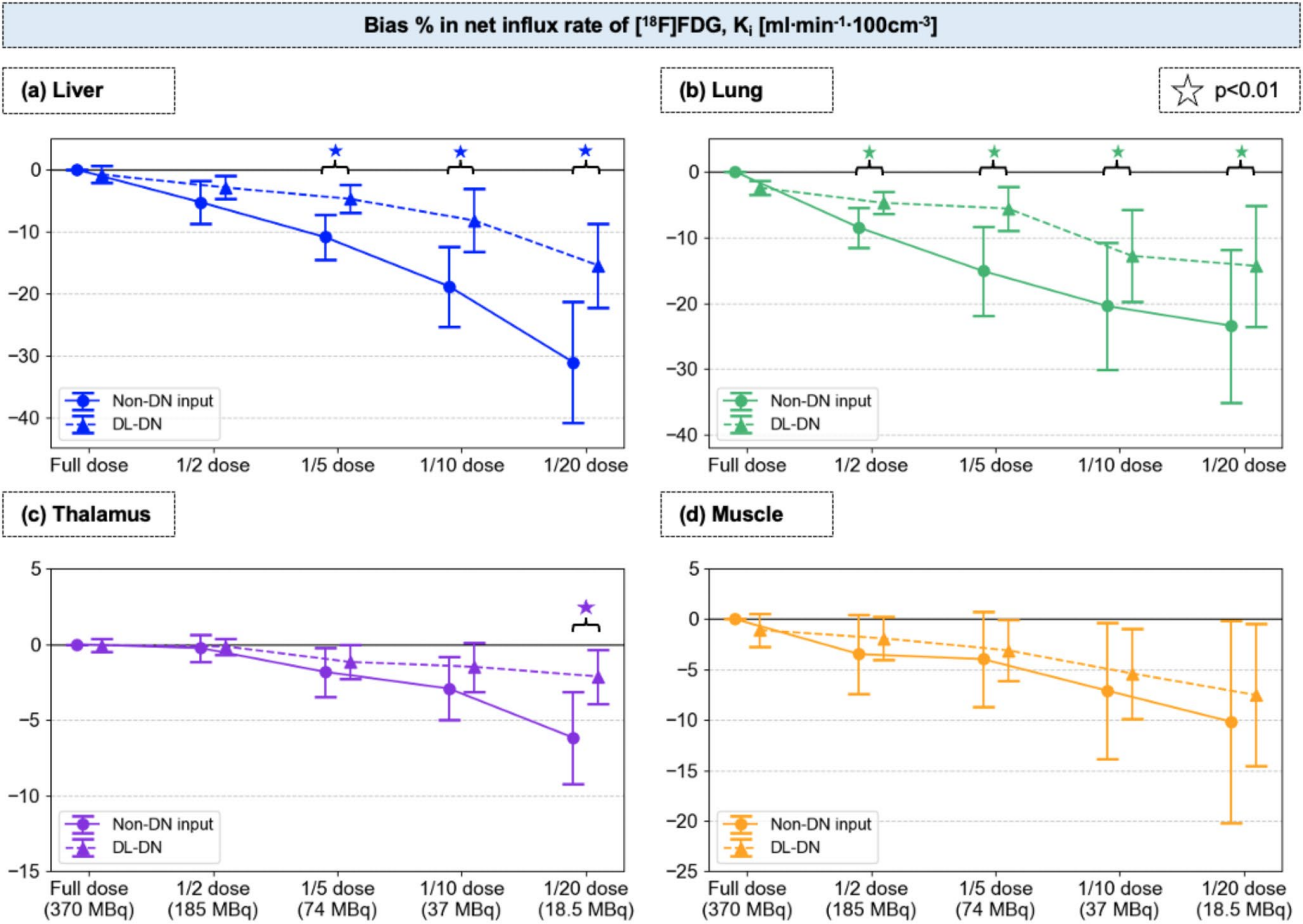
Percent bias (reported as mean and SD in 10 test subjects) in influx K_i_ rate of [^18^F]FDG from compartmental modeling for four tissues: **(a)** liver, **(b)** lung, **(c)** thalamus, **(d)** muscle. Percent bias in K_i_ is compared at five dose levels between non-DN and DL-DN. Statistical significance (*p* < 0.01) is shown for pairwise comparisons of non-DN and DL-DN (with slight off-set for visual clarity)

Figure 5 presents the mean bias and % SD in K_i_-values for the four different tissue regions. In all cases, bias becomes increasingly negative with lower doses, indicating an underestimation of K_i_, while the SD increases, reflecting greater variability due to noise. DL-DN reduces both bias and SD in K_i_ across all dose levels, improving the precision of kinetic parameter measurements. Up to a 1/5 dose reduction, the mean bias in K_i_ values with DL-DN remains below − 6%, with SD within 10%. Except for the lung, all other tissue regions exhibit a bias of less than − 10% at 1/10 dose. In the liver and lung, differences in bias in K_i_-values between non-denoised and DL-DN data are statistically significant at nearly all sub-sampled doses. At 1/20 dose, bias reaches − 31% (±9.8%) in the liver without denoising and − 15% (±6.8%) with DL-DN, while in the lung, it is -23% (±11.6%) and − 14% (±9.2%), respectively. In the thalamus, bias remains relatively low across dose levels for both non-denoised and DL-DN, with all values below − 3%, except at 1/20 dose, where the non-denoised data show a statistically higher bias (-6% ±3.1%). Compared to the other tissue regions, SD in Ki-values for the thalamus is also the smallest, with DL-DN further reducing variability. For muscle, the bias remains below − 5% until a 1/5 dose (∼ 70 MBq) for both non-denoised and DL-DN. However, at lower doses, bias becomes more negative, with no significant differences between the non-denoised and DL-DN. Similar to the observations in Fig. 4b, variability is greater in low-signal regions (lung and muscle), even with DL-DN, with SD reaching approximately − 10% starting at 1/5 dose.

**Fig. 6:**
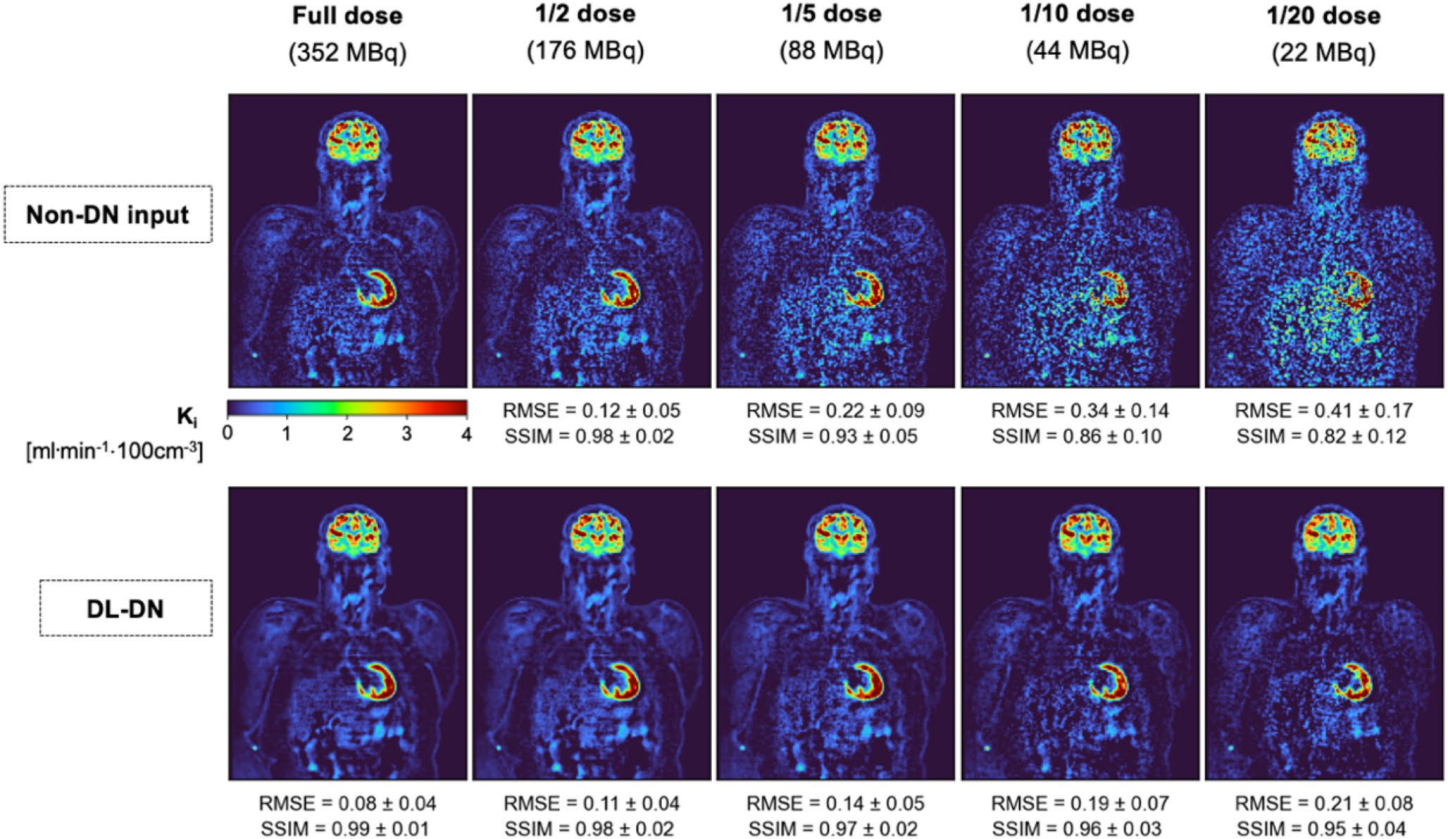
Patlak-based K_i_ parametric images (4-mm thick) from a representative subject (BMI: 35.8 kg/m^2^) who underwent a 60-min dynamic [^18^F]FDG scan are compared at five dose levels between non-DN and DL-DN. Patlak analysis was performed with t* = 25 min and the DA as IDIF. Note, the RMSE and SSIM (mean ± SD) reported in the Figure were computed for the 3D images of all 10 [^18^F]FDG test subjects with respect to their corresponding full-dose non-DN data

Figure 6 presents Patlak-based K_i_ parametric images at five dose levels for non-denoised and DL-DN, highlighting regions of low and high metabolic activity. Without denoising, substantial noise is visually evident even at 1/2 dose, degrading the quality of the parametric maps. The benefits of DL-DN are particularly apparent in regions of increased flux, with better preservation of the delineation of the left ventricular walls in this patient, even at doses as low as 1/20. Similar trends are observed in the brain, where DL-DN effectively restores structural details that are otherwise obscured by noise-dominated texture in the non-denoised images. Quantitatively, the degradation in K_i_ images at lower doses is reflected by increased RMSE and decreased SSIM values. In contrast, DL-DN stabilizes both image quality metrics across all dose levels, preserving image fidelity, even at substantially reduced doses of 1/10 and 1/20.

### Single-tracer [18F]FGln kinetic modelling

**Fig. 7:**
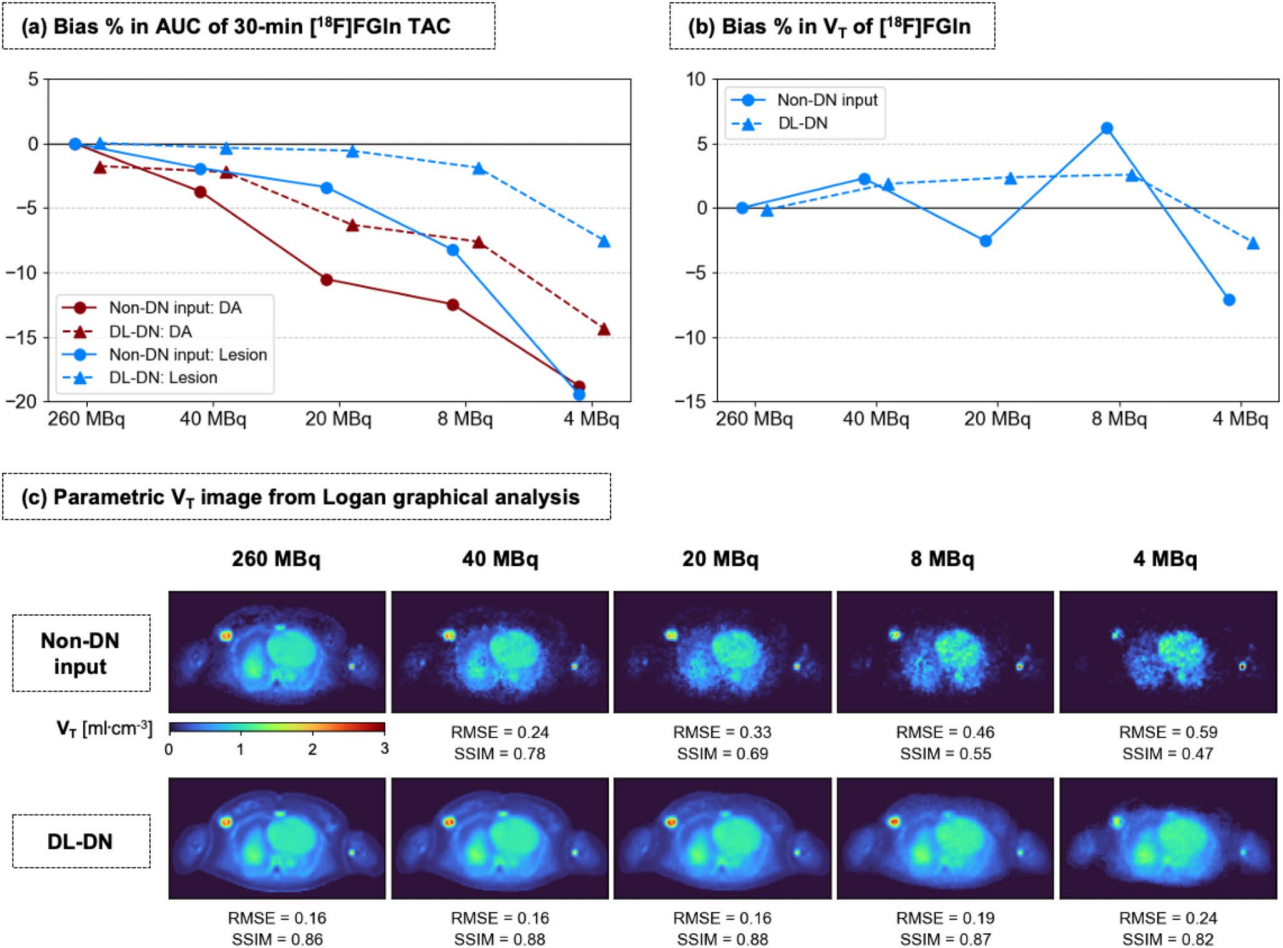
**(a)** Percent bias in AUC for the TAC of 30-min [^18^F]FGln (= one test subject), comparing results for the DA (IF, in red) and breast lesion (in blue) at five sub-sampled doses for non-DN and DL-DN (with slight off-set for visual clarity). The percent bias was calculated relative to the full-dose non-DN data. TACs are shown in SupplFig. e 3. **(b)** Percent bias in volume of distribution (V_T_) of [^18^F]FGln (from 1-TCM) for the breast lesion. **(c)** Logan-based V_T_ parametric image (t* = 2 min). RMSE and SSIM (relative to the full-dose non-DN) are reported for this 2D image slice

Figure 7a presents the bias % in [^18^F]FGln AUC of the DA and breast lesion across different dose levels. Without denoising, the AUC bias increases with decreasing dose, with the DA exhibiting a stronger deviation than the lesion, except at 4 MBq, where both regions reach about − 20%. This larger bias in the DA likely arises from uncertainties in the sharp initial peak of the IF and its subsequent rapid decline (as was also observed with the [^18^F]FDG data shown in Fig. 3a); the lesion TAC follows a more gradual time course, with a slower uptake phase and a more stable plateau. DL-DN consistently reduces the bias at all doses. For the breast lesion, the bias is mitigated from − 0.4% at 260 MBq to -7.5% at 4 MBq, compared to a bias of -19.4% at the lowest dose without denoising. Similarly, for the DA, DL-DN reduces the bias from − 1.8% to -14.3%, whereas the non-denoised bias reaches − 18% at 4 MBq.

Figure 7b presents the bias % in V_T_ of [^18^F]FGln, derived from 1-TCM analysis of the breast lesion. The non-denoised data shows considerable variation, fluctuating between positive and negative bias across dose levels, reflecting a level of uncertainty in the estimates. In contrast, DL-DN exhibits greater stability, with a systematic trend toward slightly positive bias in V_T_-values, suggesting an over-estimation relative to the reference. However, at the lowest dose (4 MBq), the bias for DL-DN shifts to negative values (within − 10%), indicating a decline in quantification accuracy.

Figure 7c displays parametric V_T_ images generated using Logan graphical analysis at five doses. The non-denoised images exhibit visible degradation at lower doses, with increased noise, reduced contrast, and loss in structural integrity. The RMSE progressively increases with decreasing dose, while the SSIM decreases, particularly below 20 MBq. Notably, in the reference image (260 MBq, non-denoised), the breast lesion shows intratumoral heterogeneity, with a lower V_T_-value in the center compared to its surroundings, indicating the presence of a necrotic tumor core. However, at lower doses, the increased presence of noise in the parametric images compromises the visibility of regions with high V_T_, leading to a loss of delineation of tumor heterogeneity. In contrast, DL-DN maintains more stable image quality across dose levels, as reflected by lower RMSE and higher SSIM, particularly at 8 and 4 MBq. With DL-DN, the tumor heterogeneity, with lower V_T_ in its center, remains visually identifiable down to 20 MBq (in this 2D slice), preserving contrast and structural details. However, at 4 MBq, the V_T_ image shows a loss in intensity for the lesion region, which is consistent with the finding in Fig. 7b, where the bias % in V_T_ for DL-DN shifts to negative value.

### Dual-tracer [18F]FGln/[18F]FDG kinetic modelling

**Fig. 8:**
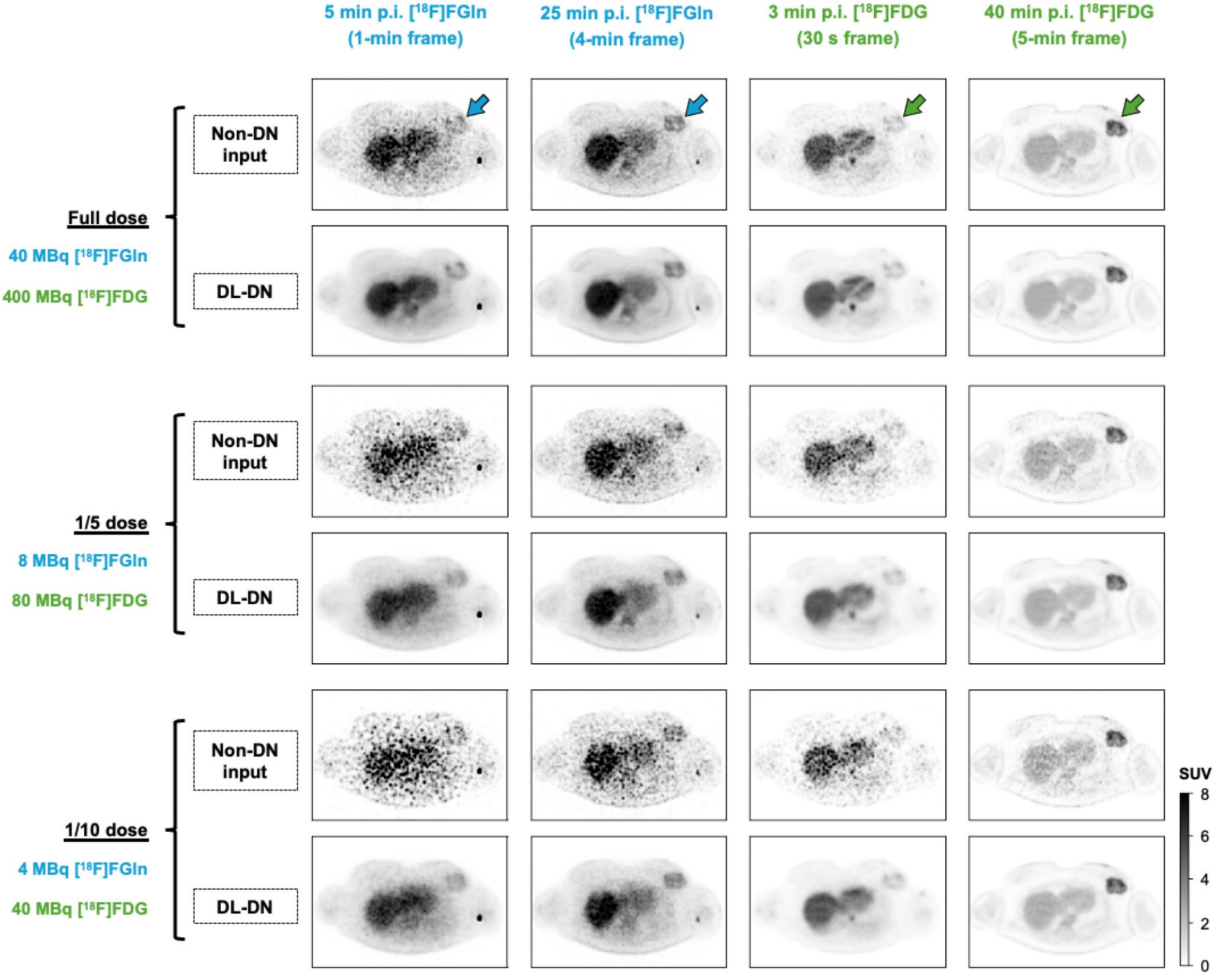
Comparison of a representative transverse image (4-mm thick) of a breast lesion shown at two time-points p.i. for [^18^F]FGln and [^18^F]FDG with different frame durations (Subject’s BMI: 39.3 kg/m^2^). Dual-tracer data which were sub-sampled to 1/5 and 1/10 of the original (“full”) dose are compared between non-DN and DL-DN. The SUV scale was normalized per tracer

The first row of Fig. 8 shows representative transverse slices at full-dose for [^18^F]FGln and [^18^F]FDG (with minimal residual[^18^F]FGln signal present as the images were obtained successively in a single imaging session) at two time-points with different frame durations. The [^18^F]FGln images are noisier than the [^18^F]FDG images, reflecting the lower injected [^18^F]FGln activity. Figure 8 further compares these images at 1/5 and 1/10 dose, without denoising and with DL-DN, which effectively reduces noise in the image frames of both tracers. DL-DN notably preserves intratumoral heterogeneity in both [^18^F]FGln and [^18^F]FDG images, better capturing the spatial variations in voxel intensities within the tumor.

**Fig. 9:**
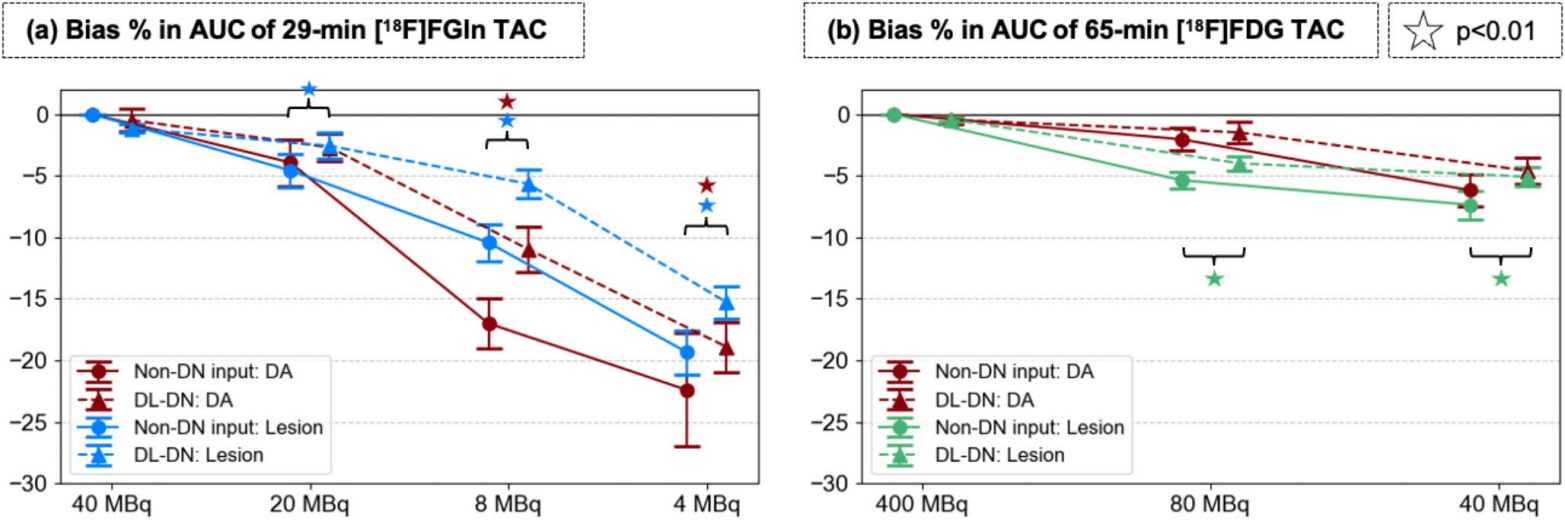
Percent bias in AUC for the TACs of **(a)** [^18^F]FGln and **(b)** [^18^F]FDG during subsequent 29-min and 65-min dynamic scans, respectively, comparing results for the DA (IF, in red) and breast lesion (in blue for [^18^F]FGln and green for [^18^F]FDG) at different sub-sampled doses for non-DN and DL-DN (with slight off-set for visual clarity). Percent bias relative to the full-dose non-DN data, reported as mean ± SD over 10 bootstrapped replicates. Statistical significance (*p* < 0.01) is shown for pairwise comparisons of non-DN and DL-DN. The corresponding TACs are shown in Suppl. Figure 4

Figure 9 presents the bias % in the AUC of the TACs for the DA and breast lesion from the [^18^F]FGln and [^18^F]FDG data at different dose levels without and with DL-DN. The corresponding TACs are compared in Suppl. Figure 4. In the initial minutes after tracer injection, noise effects on the TACs are evident as random fluctuations in SUVs and large SDs, indicated by the width of the shaded areas. Qualitatively, as seen in Suppl. Figure 4, DL-DN effectively reduces these SDs and lowers the fluctuation amplitude. Quantitatively, Fig. 9a and b confirm that DL-DN reduces both the bias and SD in AUC of TACs for the IF and lesion regions. The noise effects (i.e., larger bias values and higher SDs) are more pronounced for [^18^F]FGln, which was acquired over 29 min with an injected dose one-tenth that of [^18^F]FDG in this dual-tracer protocol. For [^18^F]FGln, at doses below 8 MBq, statistically significant differences in AUC bias are observed for both the DA and breast lesion, comparing non-denoised and DL-DN. At 20 MBq of [^18^F]FGln, biases remain below − 5%, reaching beyond − 15% at 4 MBq, even with DL-DN applied. For [^18^F]FDG, acquired over 65 min, statistically significant differences in AUC bias are found for the lesion at 80 and 40 MBq between non-denoised and DL-DN data.

**Fig. 10:**
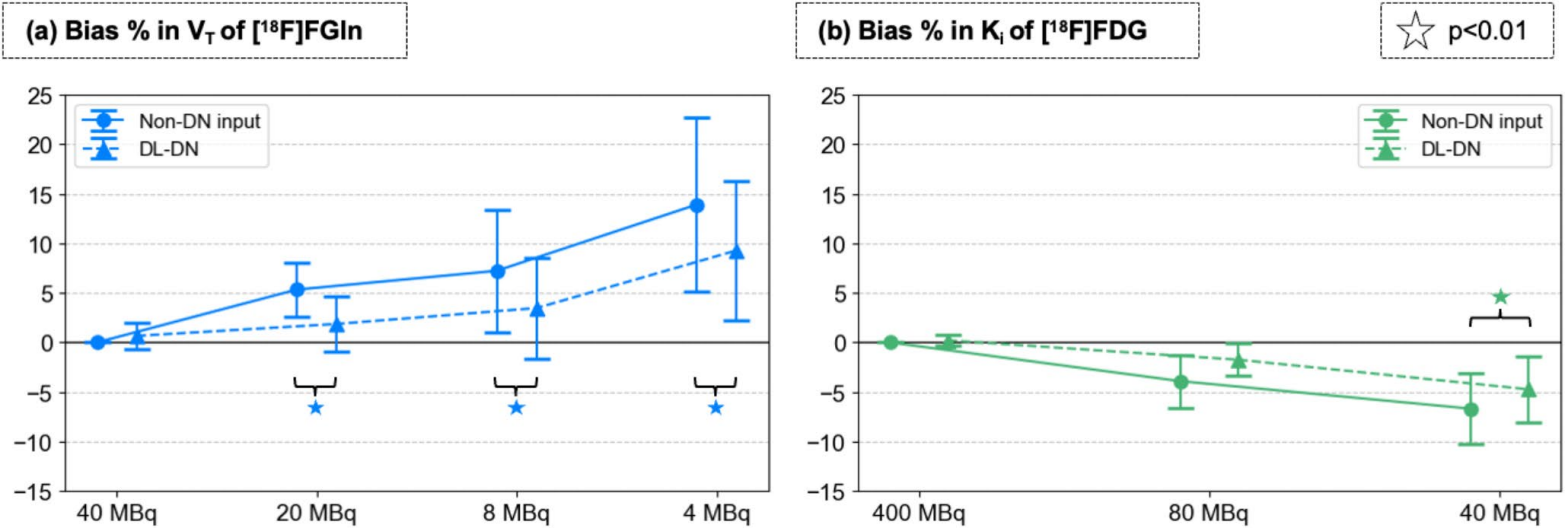
Percent bias (reported as mean and SD over 10 bootstrapped replicates) in **(a)** volume of distribution (V_T_) of [^18^F]FGln (from 1-TCM) and **(b)** influx K_i_ rate of [^18^F]FDG (from 2-TCM) for the breast lesion. Percent bias in kinetic parameters is compared at different sub-sampled doses between non-DN and DL-DN (with slight off-set for visual clarity). Note that the full-dose [^18^F]FGln for this study is equivalent to 1/10 dose of [^18^F]FDG. Statistical significance (*p* < 0.01) is shown for the pairwise comparisons of non-DN and DL-DN

Figure 10 compares the bias % in kinetic parameters estimated for the [^18^F]FGln/[^18^F]FDG dual-tracer study at different sub-sampled dose levels, without denoising and with DL-DN. For V_T_-values of [^18^F]FGln (Fig. 10a), a systematic increase toward positive bias is observed across all doses. Specifically, in the non-denoised case, the bias rises from 5.3% (±2.8%) at 20 MBq to 7.2% (±6.2%) at 8 MBq and to 13.9% (±8.7%) at 4 MBq. DL-DN mitigates this bias but follows a similar pattern, with an increase from 1.9% (±2.8%) at 20 MBq to 3.5% (±5.1%) at 8 MBq and 9.3% (±7.0%) at 4 MBq. The observed differences between non-denoised and DL-DN V_T_-values are statistically significant at all doses levels. For K_i_-values of [^18^F]FDG (Fig. 10b), a systematic shift toward negative bias is seen at reduced doses, although with a less steep decline compared to the bias in V_T_ for [^18^F]FGln. This difference may be attributed to the fact that the [^18^F]FDG doses are approximately ten times higher than [^18^F]FGln in this dual-tracer setup.

**Fig. 11:**
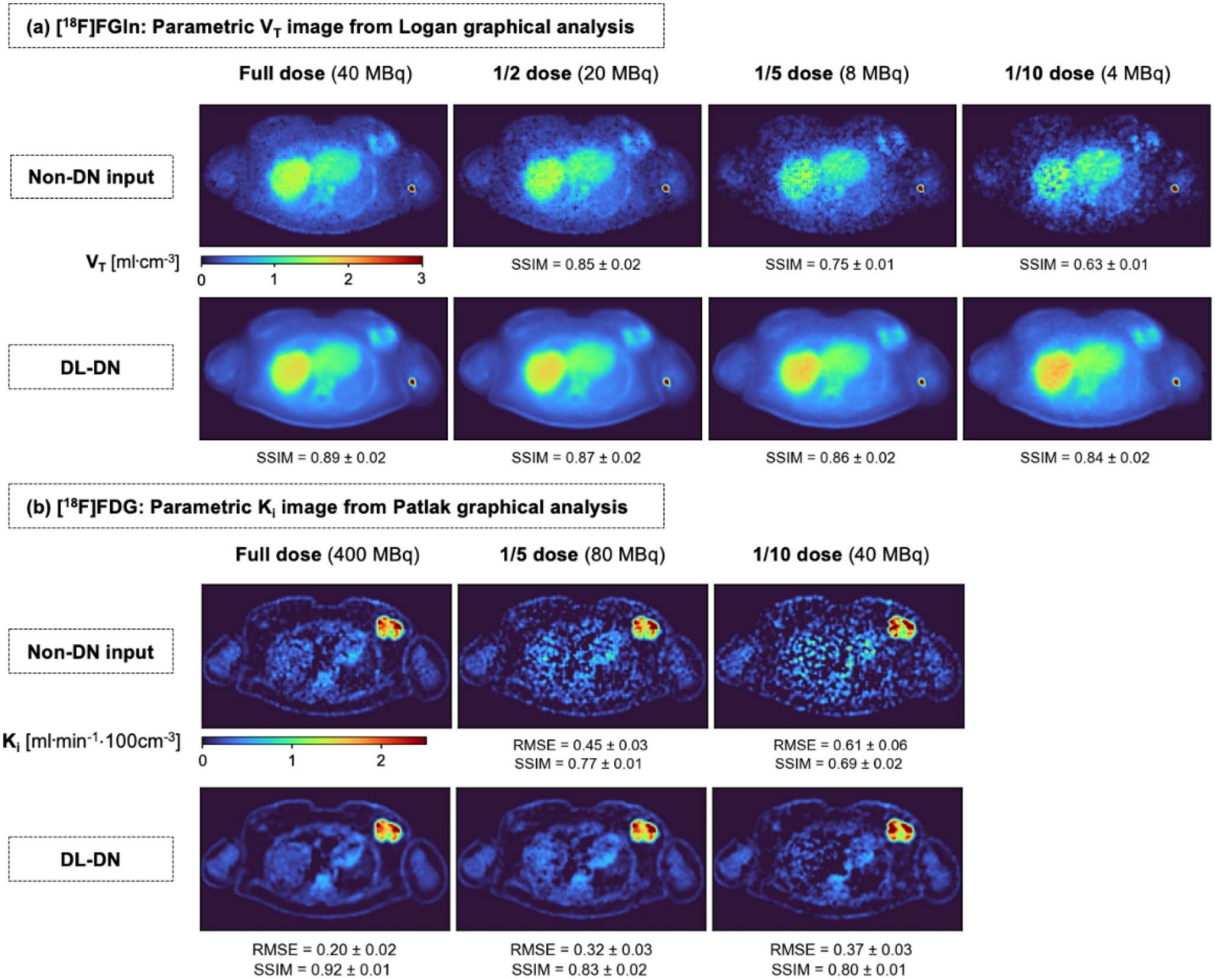
Parametric images (4-mm thick) from the dual-tracer study of a breast cancer subject (BMI: 39.3 kg/m^2^) are compared at different sub-sampled doses between non-DN and DL-DN images. **(a)** Logan-based volume of distribution (V_T_) images from 29-min [^18^F]FGln data (t* = 2 min), **(b)** Patlak-based influx rate (K_i_) images from 65-min [^18^F]FDG data (t* = 25 min). Note, the RMSE and SSIM were computed for this 2D image slice of [^18^F]FDG, while only SSIM was used for [^18^F]FGln. Results are reported as mean ± SD across 10 bootstrapped replicates

Figure 11 presents the parametric images derived from Logan graphical analysis for [^18^F]FGln (V_T_) and Patlak graphical analysis for [^18^F]FDG (K_i_) across multiple dose levels. The parametric images reveal intratumoral heterogeneity in the breast lesion, consistent with observations from Fig. 8. For the non-denoised data, the V_T_ parametric images (Fig. 11a) suffer from substantial degradation as dose decreases, with increased noise, reduced contrast, and loss of structural details, which aligns with the single-tracer [^18^F]FGln study in Fig. 7c. In contrast, DL-DN maintains a more consistent image quality (in terms of contrast and structural features) across doses, as reflected by the SSIM values. Note that we only report the SSIM values for [^18^F]FGln, as its image with the highest available dose (40 MBq) exhibits structured noise (e.g., in the liver), making a pixel-wise error metric like RMSE unreliable. Additionally, a gradual increase in V_T_-values is observed in the liver with DL-DN. For the Patlak-derived K_i_ maps of [^18^F]FDG (Fig. 11b), DL-DN effectively reduces noise while preserving finer tissue boundaries, leading to lower RMSE and higher SSIM compared to the non-denoised data.

## Discussion

Our study investigated the feasibility of applying an image-based training approach to dynamic PET. More specifically, a DL model was trained on static, single-frame [^18^F]FDG PET images from clinically relevant late time-points that were sub-sampled to various low-dose levels. This static-trained DL model was then applied for denoising multiple individual images from early to late time-points in dynamic PET studies.

### Application to dynamic single-tracer imaging: [18F]FDG and [18F]FGln

We investigated two ^18^F-labelled tracers in a single-tracer dynamic imaging setup, using a high-dose reference data to quantify bias reduction with DL-based DN for low-dose protocols. The highest administered doses were ~ 370 MBq for [^18^F]FDG and ~ 260 MBq for [^18^F]FGln. Establishing high-dose references enabled accurate quantification under various dose reduction conditions comparing non-denoised and DL-DN data. Kinetic analyses were conducted using both compartmental modeling and voxel-based graphical analysis to quantify biologically relevant parameters, including the net influx rate constant (K_i_) for [^18^F]FDG in healthy tissues, reflecting metabolic trapping, and the tissue distribution volume (V_T_) for [^18^F]FGln in breast lesions.

DL-DN significantly reduced bias and variability in AUC quantification for blood IF and tissue TACs for doses as low as 20–80 MBq. The impact varied by tissue, likely reflecting differences in uptake and clearance dynamics. DL-DN also stabilized image noise over time frame images, reducing fluctuations in TACs. Kinetic analyses indicated that bias and variability in K_i_-values increased at lower doses, with DL-DN mitigating both across all tissue regions. Liver and lung showed significant differences in K_i_-values between non-denoised and DL-DN data for doses below 40 MBq. The thalamus showed minimal bias, whereas muscle exhibited increasing bias at very low doses, with no significant DL-DN effect. These findings suggest that several factors influence parameter accuracy, including count statistics (dose level), region size (e.g., thalamus vs. liver or lung), and target uptake relative to the background (e.g., low-signal regions like lung and muscle vs. high-signal regions like liver and thalamus). Viswanath et al. [8] investigated bias of kinetic parameter quantification for low-, medium-, and high-flux lesions using a sphere-embedding study. They reported more accurate parameter estimation for high-flux spheres, attributing this to higher uptake at early time points, which improves measurement accuracy and reduces susceptibility to noise. This aligns with our findings that low-signal regions, such as the lung and muscle, exhibited greater SD under reduced-dose conditions. The bias results with DL-DN suggest that more reliable kinetic parameter estimates can be obtained over a larger dose range.

This study focused on assessing the impact of DL-DN on the quantitative accuracy of estimating influx rate (K_i_) for [^18^F]FDG and distribution volume (V_T_) for [^18^F]FGln under varying noise levels. Denoising methods can affect the peak of the IF and, in turn, influence estimation of parameters sensitive to early-phase dynamics, such as K_1_, which represents the rate of tracer delivery from blood to tissue. For completeness, the bias % (mean ± SD) in K_1_ across 10 [^18^F]FDG test subjects are included in supplemental material (Suppl. Figure 5). These results​ indicate that while DL-DN reduces the K_1_ bias and SD in comparison to the non-denoised data, considerable variability remains, and no clear systematic trend is observed across reduced doses. Parameters such as fractional blood volume and blood delay, which are estimated during kinetic model fitting, are known to influence K_1_​ estimates and may contribute to the observed variability, particularly in low statistics data. Viswanath et al. [8] reported that allowing the blood fraction to be fitted in kinetic modeling improved K_i​_ bias in the liver but increased K_1_ bias to 50–100%. In a study by Chen et al. [50], investigating [^13^N]ammonia perfusion tracers, they showed that reconstruction algorithms and smoothing techniques can introduce quantification biases in K_1_. Li et al. [51] also showed that K_1_ were underestimated when delay was not included in the fitting process. This highlights the need for further investigation into the sources of K_1_ variability. Future work will involve detailed sensitivity analyses (including fixing vs. fitting specific parameters) to assess the impact on these estimates of different low-dose conditions and denoising algorithms.

Of note is the use of training and testing subject data from the high-sensitivity LAFOV PennPET Explorer (142 cm AFOV). The increased sensitivity of long AFOV PET has been shown to improve the quantitative accuracy of parametric estimates compared to standard AFOV scanners (< 30 cm) [6, 8, 25]. These systems also facilitate IDIF measurements, as large blood pools are contained in the same FOV as tissues of interest. Despite improved temporal sampling, total-body parametric imaging remains challenging due to noise amplifications at the voxel level, which may lead to numerical identifiability issues and higher susceptibility to local minima, further compromising the reliability of quantitative measurements [2, 7]. This study demonstrated that DL-DN can improve the data quality of dynamic PET (i.e., reduced noise in the time frame images and improved TACs), resulting in the generation of higher-quality parametric images of K_i_ (for [^18^F]FDG) and V_T_ (for [^18^F]FGln) obtained by voxel-based Patlak and Logan graphical analyses, even at substantial dose reductions down to 20 MBq. In contrast to the substantially noisier parametric images without denoising at decreasing dose levels, DL-DN was shown to stabilize RMSE and SSIM metrics across various dose conditions. The preservation of tumor heterogeneity in Logan V_T_ images (Fig. 7c) and finer delineation of high-flux regions in Patlak K_i_ images (Fig. 6) suggest that DL-DN maintains critical image features that are particularly relevant for applications in oncology, where parametric imaging can provide insights into tumor biology. The combination of the increased sensitivity afforded by the PennPET Explorer [17] and application of DL-DN allows for quantitatively accurate parametric imaging at low administered activity, down to 20–40 MBq, that would be especially desirable in certain patient populations.

### Application to dynamic dual-tracer imaging: [18F]FGln/[18F]FDG

The performed dual-tracer protocol leveraged the high sensitivity of the PennPET Explorer to use a lower initial dose of [^18^F]FGln (40 MBq) followed by higher dose of [^18^F]FDG (400 MBq). Optimal tracer dosage and timing of each injection was determined through sub-sampling studies with single-tracer scan protocols, aiming to ensure accurate kinetic parameter estimation for both tracers while minimizing the impact of residual [^18^F]FGln signal after FDG injection [30, 31]. In this study, we evaluated the potential for further dose reduction using DL-DN by analyzing data with both tracer doses reduced down to 1/10 of their original values. Qualitatively, DL-DN improved the image quality of both tracers across the imaging interval and preserved intratumoral heterogeneity in the breast lesion, as also supported by the comparison in parametric image quality (Fig. 11). The maintained spatial variations in voxel intensities within the tumor are likely important for texture analysis which offers additional predictive and prognostic insights [52, 53].

DL-DN mitigated noise-induced bias and variability in kinetic parameter estimates for both tracers, although its effectiveness differed between [^18^F]FGln and [^18^F]FDG due to differences in injected dose, resulting in much noisier dynamic [^18^F]FGln data as input. Specifically, full-dose [^18^F]FGln was equivalent to 1/10 of the injected activity of [^18^F]FDG, meaning that the 1/10 dose of [^18^F]FGln was roughly equivalent to 1/100 of the [^18^F]FDG dose. For [^18^F]FDG, DL-DN maintained accurate Ki quantification across all dose levels, even at an order-of-magnitude dose reduction. This aligns with our above findings from the dynamic [^18^F]FDG PET study, which showed that DL-DN maintained K_i_ accuracy in high- and low-signal tissue regions. For [^18^F]FGln, DL-DN was less effective at fully compensating for the pronounced noise at very low doses (< 8 MBq). While it reduced bias in V_T_ estimates and resulted in statistically lower bias values compared to the non-denoised data, a systematic increase toward positive bias, indicating V_T_ overestimation, persisted as dose decreased. Recall for the single-tracer [^18^F]FGln data, there was a sudden shift toward negative bias at 4 MBq, suggesting a threshold beyond which uncertainty increases and accuracy deteriorates, likely due to severe noise effects that the DL-DN cannot recover. The parametric images further highlighted this, showing that although DL-DN improved structural integrity and contrast, it did not entirely mitigate the degradation, specifically for the breast lesion, observed in the non-denoised low-dose [^18^F]FGln data. Overall, for the dual-tracer study, bias trends in AUC and kinetic parameters were consistent with single-tracer findings, with good agreement between K_i_ and V_T_ trends from compartmental modeling and the qualitative assessment of the lesion in parametric images.

This work presents a first attempt to use a DL-DN model in a dual-tracer PET study. Traditionally, the robustness of dual-tracer methods was limited by the lower sensitivity of standard AFOV systems, which led to low-count statistics and required higher activities to be injected for both tracers to achieve accurate parameter estimates. This imposed waiting for sufficient radioactive decay between scans, often resulting in separate imaging sessions (e.g., a day apart with [^18^F]). Utilizing a LAFOV PET enabled the dual-tracer approach utilized here, with a low dose of the first tracer followed by a greater dose of the second to overwhelm the first. Advanced denoising algorithms, as studied here, offer additional promise to further optimize, and expand, these protocols. In particular, such DL approaches may enable similar low-high dose protocols on standard AFOV PET that do not benefit from such sensitivity gains as LAFOV PET. We applied the DL-DN model in a dual-tracer study of two radiotracers labeled with the same isotope, each quantifying different aspects of tumor metabolism, but this approach could be extended to other tracer combinations of varying kinetics and different relative uptakes.

### Static-trained neural network and future optimizations

The proposed DL-DN network, trained on static [^18^F]FDG PET images, addresses count statistic differences between early and late frames by incorporating various count reduction levels, but it does not account for the temporal variations in tracer uptake inherent in dynamic PET. This limitation may affect the denoising performance of the current framework: early frames often exhibit high local count density due to concentrated tracer uptake in specific regions (blood pool), but have low overall count statistics due to shorter frame durations. In contrast, later frames tend to show a more uniform tracer distribution throughout the body. Further network optimizations may be needed to enhance denoising performance across a wider range of activities for tracers of different uptake characteristics (e.g., [^18^F]FGln has a faster clearance than [^18^F]FDG).

To move beyond the current approach of treating denoising as a purely local task independent of count density and distribution profiles, we aim to optimize the DL-DN approach for enhanced 4D denoising performance by incorporating both spatial and temporal information, potentially through “distribution-aware” networks that leverage temporal correlations across sequential image frames. Previous work in this area include Chen et al. [54] who proposed denoising in the temporal domain by smoothing TACs at the pixel level, instead of denoising dynamic images. De Benetti et al. [55] developed a spatio-temporal U-Net to generate parametric images from dynamic PET data, using a self-supervised loss formulation to ensure similarity between TACs measured from the data and those determined via kinetic modelling. Future work will also include a comparison with alternative denoising methods, such as the kernel method [19–21].

## Conclusions

This study demonstrated the feasibility and benefits of applying a DL-DN framework, trained on static [^18^F]FDG PET images, to decrease injected dose on dynamic PET studies. DL-DN effectively reduced image noise, improved consistency in tissue and blood IF TACs, and systematically reduced tissue-dependent bias and variability in K_i_ and V_T_ down to 40 MBq doses, while considerably enhancing image quality for both Logan V_T_ and Patlak K_i_ parametric images. For the [^18^F]FGln/[^18^F]FDG dual-tracer study, DL-DN preserved accurate K_i_-values of the breast lesion even at a ten-fold dose reduction (40 MBq) and resulted in statistically lower bias in V_T_ values compared to non-denoised data. Results showed that dose reductions, up to 5-fold, were possible with DL-DN while maintaining quantitative accuracy in kinetic parameter estimates. In conclusion, DL-DN presents promise in improving quantification for dynamic PET imaging at low-doses. Such methods can reduce radiation exposure for routine single tracer studies as well as facilitate novel protocols, such as dual-tracer PET.

## Electronic supplementary material

Below is the link to the electronic supplementary material.

Supplementary Material 1

## Data Availability

The datasets generated during and/or analyzed during the current study are available from the corresponding author on reasonable request.
