# Supplementary material for: Impact of deep learning denoising on kinetic modelling for low-dose dynamic PET: application to single- and dual-tracer imaging protocols
Source: Eur J Nucl Med Mol Imaging. 2025 Mar 12;52(9):3465–83. doi: 10.1007/s00259-025-07182-6 (PMC12222255; doi:10.1007/s00259-025-07182-6)
Supplement: Supplementary file 1 — Supplementary Material 1 [file 259_2025_7182_MOESM1_ESM.pdf]

Supplementary Information: "Impact of Deep Learning Denoising on Kinetic Modelling for Low-Dose Dynamic PET: Application to Single- and Dual-Tracer Imaging Protocols"

Journal: European Journal of Nuclear Medicine and Molecular Imaging

Authors:

Florence M Muller[1,2], Elizabeth J Li[2], Margaret E Daube-Witherspoon[2], Austin R Pantel[3], Corinde E Wiers[4], Jacob G Dubroff[3], Christian Vanhove[1], Stefaan Vandenberghe[1], Joel S Karp[2]

	16-digit ORCID
Florence M Muller[1,2]	0000-0001-5287-7355
Elizabeth J Li[2]	0000-0003-2365-0594
Margaret E Daube-Witherspoon[2]	0000-0002-6318-2054
Austin R Pantel[3]	0000-0001-8649-9970
Corinde E Wiers[4]	0000-0002-2934-8794
Jacob G Dubroff[3]	0000-0002-0732-2374
Christian Vanhove[1]	0000-0002-3988-5980
Stefaan Vandenberghe[1]	0000-0002-2377-3968
Joel S Karp[2]	0000-0002-6154-8585

Affiliations:

1 Medical Image and Signal Processing, Faculty of Engineering and Architecture, Ghent University, Ghent, Belgium

2 Physics and Instrumentation, Department of Radiology, University of Pennsylvania, Philadelphia, United States

3 Division of Nuclear Medicine Imaging and Therapy, Department of Radiology, University of Pennsylvania, Philadelphia, United States

4 Center for Studies of Addiction, Department of Psychiatry, University of Pennsylvania, Philadelphia, United States.

Corresponding author:

Florence M Muller

florencemarie.muller@ugent.be

Suppl. Material (1) **Comparison of Count Statistics between Static Sub-sampled Data (Training) and Dynamic Frame Data (Testing)**

Static [^{18}F]FDG PET images were reconstructed at a late time-point p.i. (e.g., 50-60 mins p.i.) and sub-sampled with seven reduction factors (from 1/2 down to 1/300 of original counts). These sub-sampled images were used to train seven supervised denoising neural networks, each corresponding to a specific count level. The resultant "static-trained" DL-DN model was applied for frame-by-frame denoising of dynamic PET data, reconstructed as multiple frames at different time-points and of varying durations. Each frame image was denoised by the network trained on the static condition that most closely matched its count statistics.

Suppl. Figure 1 illustrates the total counts in the global image for the "static" low-dose levels (represented as grey bar graphs) in comparison to the total counts from the dynamic frame data (depicted as horizontal dotted lines, color-coded by frame duration). This visualization highlights the correspondence between the static (single-frame) training data and the dynamic testing data, demonstrating that the sub-sampled dose levels used for network training adequately encompass the range of count statistics observed in the dynamic testing data.

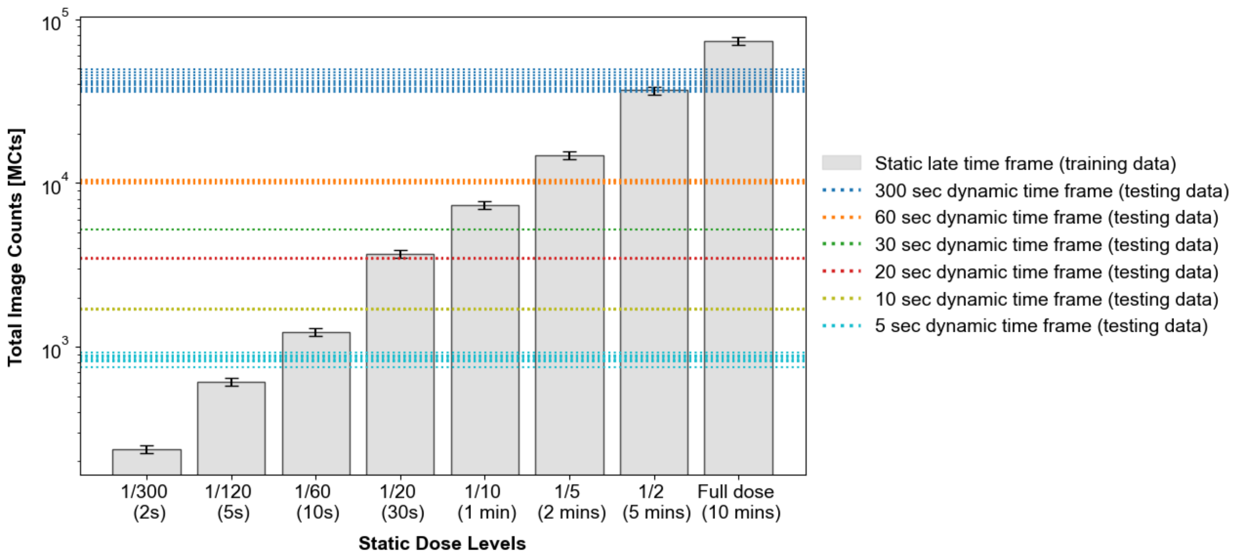

Suppl. Figure 1: Comparison of total image counts between static sub-sampled data (training) and dynamic frame data (testing). The bar graphs show the count statistics for the static late frame data across the different dose levels used in training. The bar height represents the average image counts across the 13 datasets from the pilot study on alcohol use disorder. The horizontal dashed lines represent the image counts in the dynamic time frames, with each line corresponding to the count statistics for a specific frame duration. The different frame durations (5s, 10s, 20s, 30s, 60s, 300s) are color-coded for clear differentiation. Note, logarithmic scale is used on the y-axis to accommodate the wide range of image counts.

Suppl. Material (2) **<u>Structure of the 2D U-Net with Attention Gates</u>**

(a) Neural network architecture

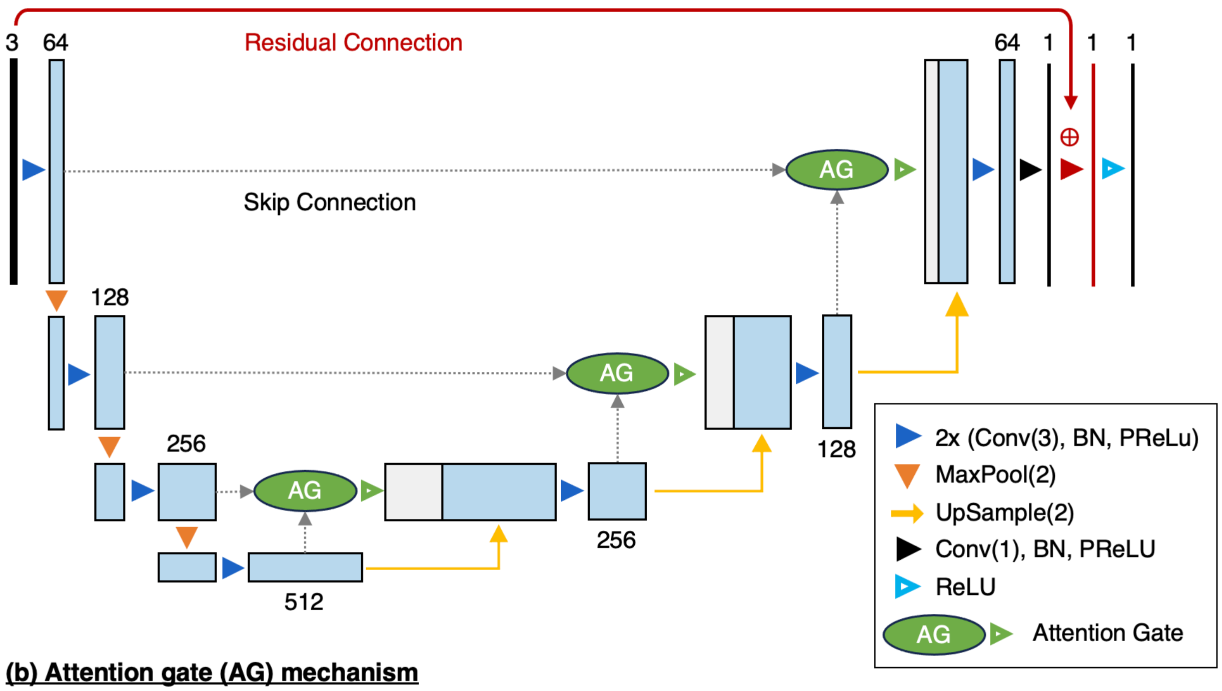

(b) Attention gate (AG) mechanism

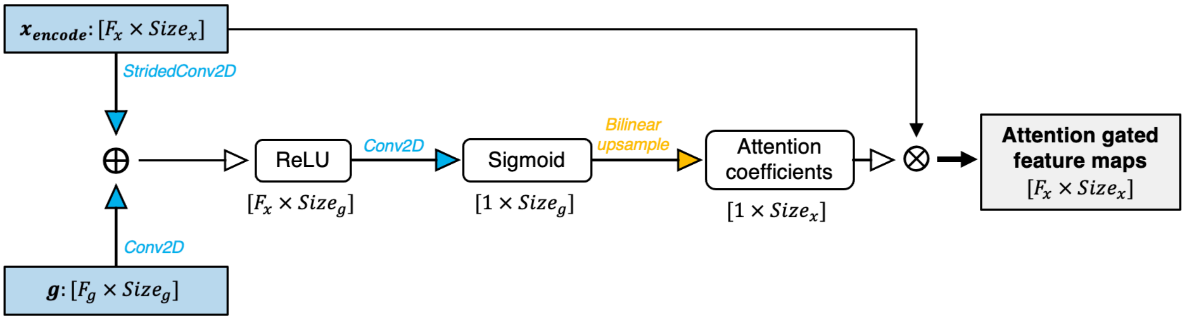

Suppl. Figure 2: (a) Schematic of the implemented U-Net structure with attention gates (AG) to filter the encoder feature maps propagated through the skip connections. The number of trainable parameters is around 11.2 million. **(b)** Illustration of the AG mechanism (adopted from Oktay O *et al.* [1]) that scales the feature maps from the encoding part with attention coefficients computed in the AG module. The attention-gated feature maps are then concatenated with the output of the up-sampling layer in the decoder.

[1] Oktay O, Schlemper J, Folgoc LL, Lee M, Heinrich M, Misawa K, et al. Attention u-net: Learning where to look for the pancreas. arXiv preprint arXiv:180403999. 2018

Suppl. Material (3) Comparison of TACs for Dynamic Single-Tracer [^{18}F]FGln

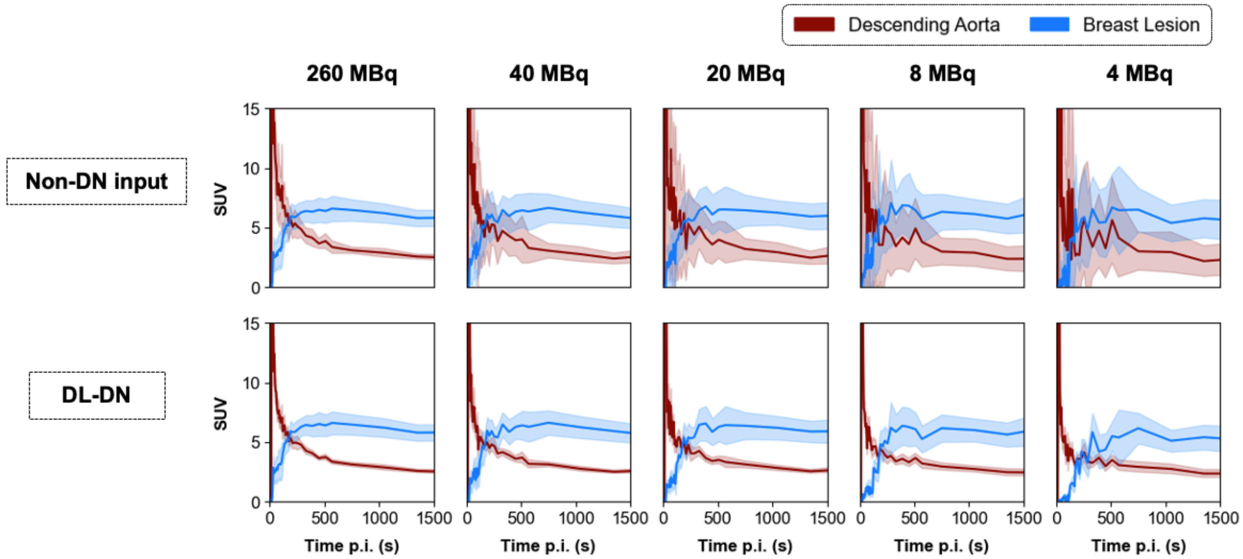

Suppl. Figure 3: TACs of the DA (IF, in red) and breast lesion (in blue) show the kinetics of [^{18}F]FGln during 30-mins dynamic scan, comparing five sub-sampled doses for non-denoised (non-DN) and DL-DN data from a single replicate. Solid lines represent the SUV_{mean} in each VOI with shaded areas indicating the SD in SUV (as noise measure).

Suppl. Material (4) **<u>Comparison of TACs for Dynamic Dual-Tracer [^{18}F]FGln/[^{18}F]FDG</u>**

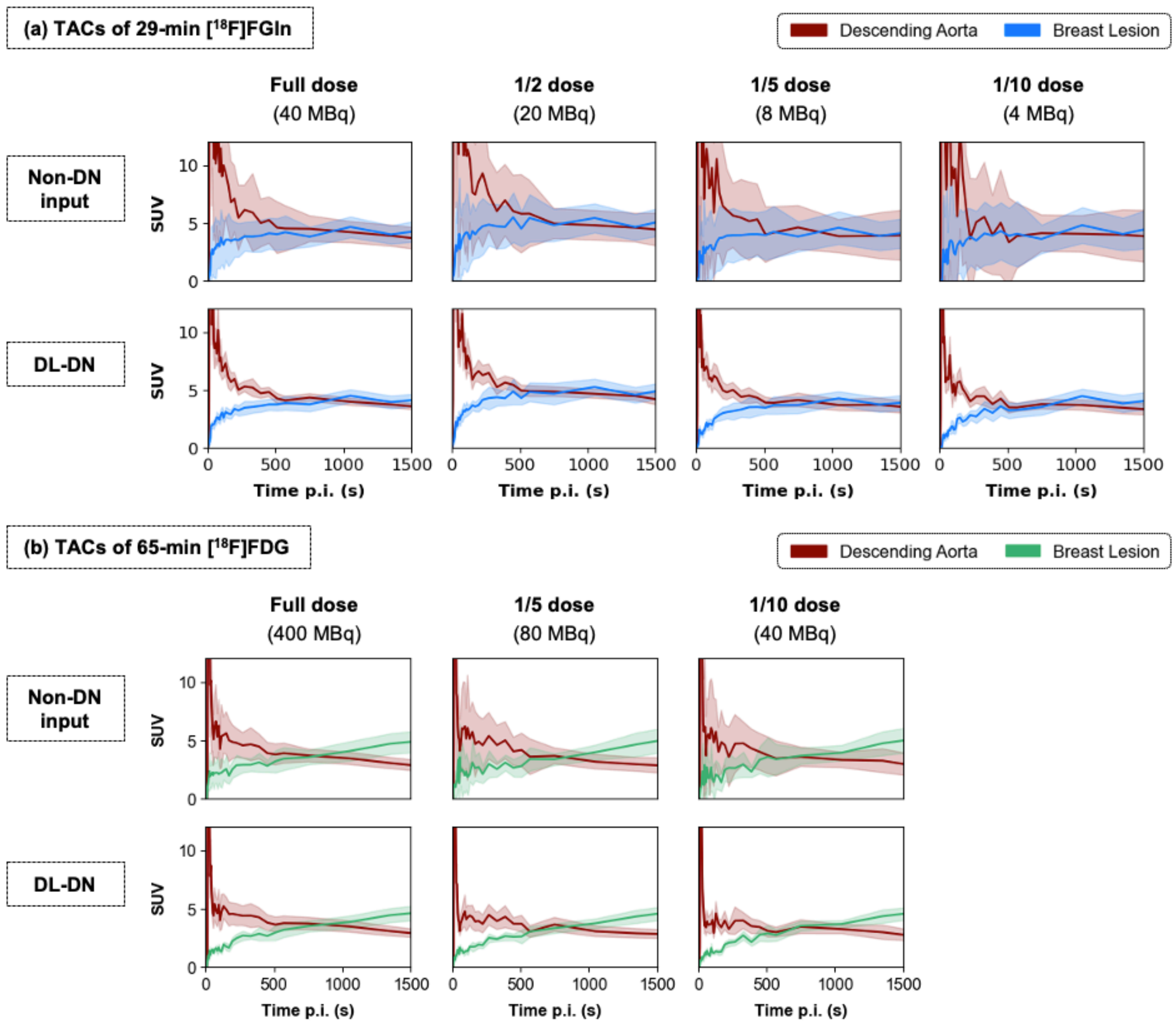

Suppl. Figure 4: TACs of the DA (IF, in red) and breast lesion (in blue and green) show the kinetics of **(a)** [^{18}F]FGln and **(b)** [^{18}F]FDG during subsequent 29-min and 65-min dynamic scans, respectively, comparing different sub-sampled doses for non-denoised (non-DN) and DL-DN data from a single replicate. Solid lines represent the SUV_{mean} in each VOI with shaded areas indicating the SD in SUV (as noise measure). The SUV scale was normalized per tracer.

Suppl. Material (5) **Comparison of Bias % in K1 of [^{18}F]FDG**

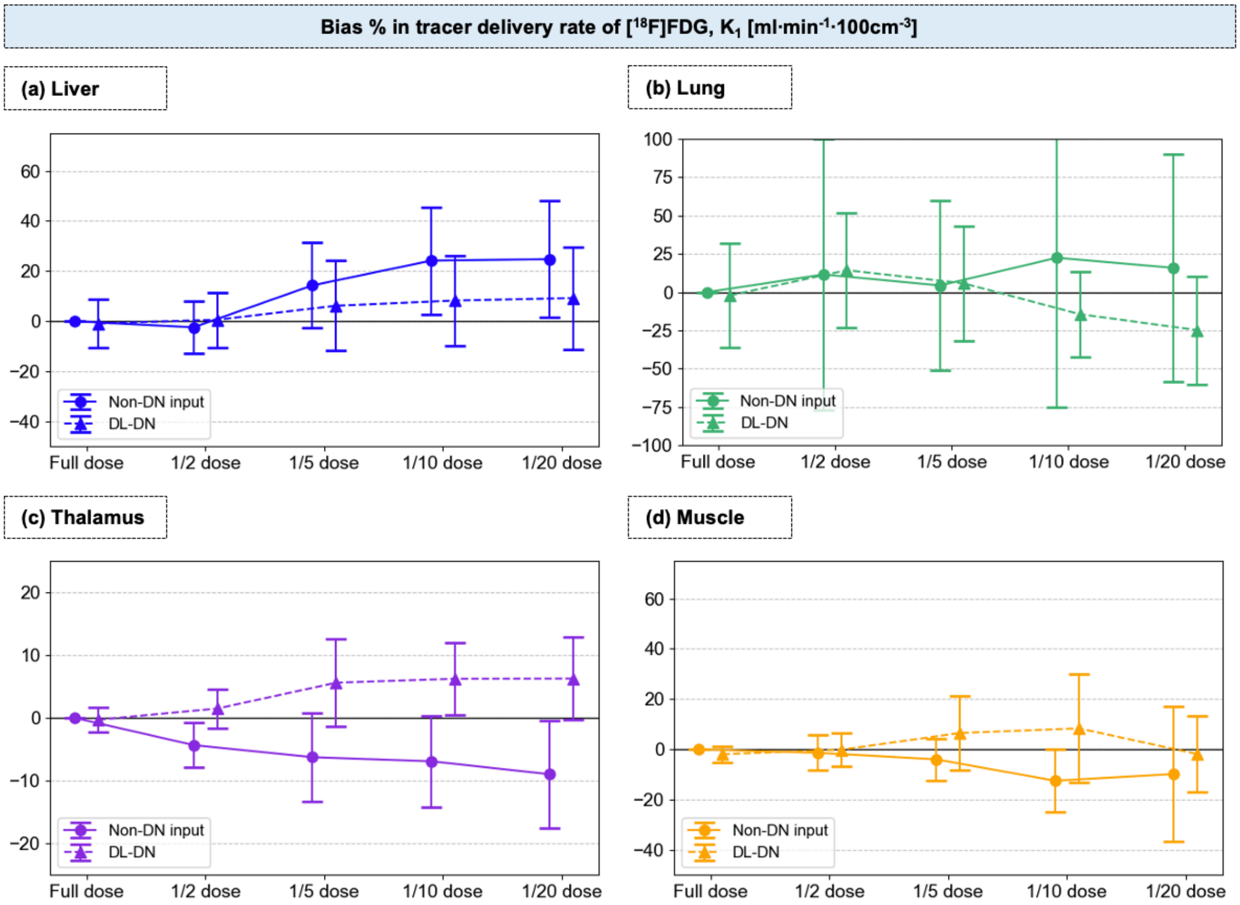

Suppl. Figure 5: Percent bias (reported as mean and SD in 10 test subjects) in tracer delivery K_1 rate of [^{18}F]FDG from compartmental modeling for four tissues: **(a)** liver, **(b)** lung, **(c)** thalamus, **(d)** muscle. Percent bias in K_i is compared at five dose levels between non-denoised (non-DN) and DL-DN.
